# Personal, social and environmental correlates of vegetable intake in normal weight and overweight 9 to 13-year old boys

**DOI:** 10.1186/1479-5868-3-37

**Published:** 2006-10-25

**Authors:** Ilse De Bourdeaudhuij, Agneta Yngve, Saskia J te Velde, Knut-Inge Klepp, Mette Rasmussen, Inga Thorsdottir, Alexandra Wolf, Johannes Brug

**Affiliations:** 1 Department of Movement and Sport Sciences, Ghent University, Ghent, Belgium; 2 Unit for Preventive Nutrition, Karolinska Institute, Stockholm, Sweden; 3 Erasmus University Medical Center Rotterdam, Department of Public Health, Rotterdam, The Netherlands; 4 Department of Nutrition, Faculty of Medicine, University of Oslo, Oslo, Norway; 5 Department of Social Medicine, University of Copenhagen, Copenhagen, Denmark; 6 Unit for Nutrition Research, Landspitali University Hospital, Reykjavik, Iceland; 7 Institute of Nutrition, University of Vienna, Austria

## Abstract

**Background:**

The first aim of the present study was to investigate differences in correlates of vegetable intake between the normal weight and the overweight boys in the Pro Children Cross Sectional Study. The second aim was to explore whether the association between vegetable intake and potential correlates is different in overweight boys compared with normal weight boys.

**Methods:**

Random samples of mainly 11-year old children were recruited in 9 European countries. The total sample size consisted of 3960 boys (16.5% overweight). A validated self-report questionnaire was used to measure vegetable intake, and personal, social and environmental factors related to vegetable intake in the classroom. Weight and height were reported by the parents of the children in parents' questionnaires.

**Results:**

Regression analyses explained 23% to 28% of the variance in vegetable intake by potential correlates. Liking, self-efficacy and bringing vegetables to school were related to intake in both normal weight and overweight boys (β's>0.10). Active parental encouragement and availability at home was only related to intake in overweight boys (β's>0.10), whereas knowledge about recommendations was only related to vegetable consumption in normal weight boys (β>0.10)

**Conclusion:**

Intervention strategies to increase vegetable intake should focus on increase in liking and preferences, increase in self-efficacy, and increase in bringing vegetables to school in both normal weight and overweight boys. Further research should investigate whether advising parents of overweight boys to encourage their child to eat vegetables every day, to insist as far as possible that their child eats vegetables regularly and to make vegetables easily available at home is effective in changing vegetable intake.

## Background

The prevalence of overweight and obesity in children and adolescents is increasing worldwide [1,2]. Overweight is caused by a long-term energy imbalance with energy intakes exceeding energy requirements for normal growth and physical activities. Promotion of a better energy balance is aimed at diet and physical activity changes. One possible strategy to prevent unnecessary weight gain in youth is promotion of fruit and vegetable consumption, since most fruits and vegetables are foods with low energy density [3-5]. Two reviews were executed recently on the relationship between weight management and fruit and vegetable intake based on epidemiological studies [4] and intervention studies [5]. In adults higher fruit and vegetable intake was associated in most studies with lower BMI. Intervention studies showed that advice to increase consumption of fruit and vegetables was related to weight loss, possibly through enhancing satiety and avoiding feelings of deprivation and hunger. More consistent weight loss and weight maintenance was found when the advice to increase fruit and vegetable consumption was combined with the advice to decrease fat intake and/or total energy intake. In children, very few studies looked at this relationship. Two epidemiological studies found that higher vegetable intake among boys was associated with lower BMI z-scores. One cross-sectional study also found that overweight boys and girls ate less fruit [4].

Since intakes of fruit and vegetables in children had not extensively been monitored on an international level, the Pro Children consortium recently collected unique data on fruit and vegetable intakes of 11-year old children in nine European countries (Austria, Belgium, Denmark, Iceland, The Netherlands, Norway, Portugal, Spain, Sweden) [6]. In a parallel parent questionnaire, data on height and weight of the children were collected. Yngve et al. [7] investigated associations between overweight/obesity and fruit, fruit juice and vegetable intake in these children across Europe to explore if earlier reports on inverse associations between fruit and vegetable intake [8] and BMI could be confirmed. Results for the total sample (n = 8317) showed that daily vegetable intake was associated with lower chances of being overweight in boys (Odds Ratio = 0.83, C.I. = 0.70–0.99), but not in girls (Odds Ratio = 0.91, C.I. = 0.76–1.09). However, the associations observed in the different countries were more diverse. Only in one country, Belgium, the association was significant, in another three of the nine countries, Austria, Iceland, and Norway, the Odds Ratios predicting normal weight/overweight from vegetable intake in boys were lower than 1 and also lower than the Odds Ratio for the total sample of boys (OR ranging from 0.41 – 0.76) [7]. The association of higher vegetable intake among boys with lower BMI is in line with the few epidemiological studies in children showing only an association in boys and not in girls [4].

In the present manuscript we therefore explored potential determinants of vegetable intake in the 9 to 13-year old boys participating in the Pro Children Cross Sectional Study and its relation to overweight. A detailed investigation of potential personal, social and environmental factors related to vegetable intake is warranted because they can be targeted as mediating variables in intervention programs. A number of studies have already investigated psychosocial and environmental correlates of food in general [9] and vegetable intake in children [10]. An earlier study [11,12] based on the Pro Children data showed that daily vegetable intake was mainly associated with knowledge of the national recommendations, positive self-efficacy, positive liking and preference, and parental modeling and demand. Contrary to the results for fruit intake, where these factors were associated fairly consistently with daily fruit intake across all nine European countries, the pattern was, however, less consistent for vegetable intake. Cultural differences between countries might be responsible for this larger diversity [13].

However, no studies have investigated whether differences in these correlates exist between overweight and normal weight children and whether their association with intake is different for overweight and normal weight children. Understanding the differences between overweight and normal weight boys on these factors may provide crucial information in designing effective interventions. If correlates of vegetable intake are dependent on weight status (overweight versus normal weight) in predicting vegetable intake, the tailoring of interventions to increase vegetable intake specific to these correlates in overweight youngsters is warranted.

The first aim of the study was to investigate differences in correlates of vegetable intake between the normal weight and the overweight boys in the Pro Children Cross Sectional Study. It was hypothesized that the overweight boys would perceive less positive general attitudes towards vegetables, less benefits, less social support, lower self-efficacy and more barriers related to vegetable intake. The second aim was to explore whether the prediction of vegetable intake by the personal, social and environmental correlates is different in normal weight and overweight boys. Analyses were conducted among the total sample as well as in the sub-sample of the 4 countries with a clear negative association between low vegetable intake and overweight or obesity.

## Methods

### Sample

The sample was from the Cross-Sectional Study of the Pro Children project [6]. This project aimed at promoting and sustaining health through increased vegetable and fruit consumption among European schoolchildren, involving nine European countries (Austria, Belgium, Denmark, Iceland, the Netherlands, Norway, Portugal, Spain and Sweden). The cross-sectional survey was conducted during October – December 2003. Pupils completed a questionnaire in the classroom. Ethical approval was obtained from all relevant ethics committees in all countries and written informed consent forms were signed by parents of all participating children.

Schools constituted the sampling unit, and from each country random samples of 11-year old children were recruited. A participation rate of 90.4% was reached in the participating schools; mean age was 11.4 years (range 8.8–13.8, SD = 0.48; 79 % of the children was born in 1992). The final sample sizes varied from 1105 for the Netherlands to 2134 for Portugal, with a total sample size of 13305. A detailed description of the Pro Children project, including the sampling and data collection procedure is given elsewhere [8,14].

### Questionnaire

A self-report questionnaire was developed to measure fruit and vegetable intake, and related correlates. The development of the questionnaire was based on theoretical models, a literature review, focus group interviews with children, individual interviews with parents and school staff, and thorough pre-testing [10,11,15]. The questionnaire included 15 constructs that were analogous for fruit and for vegetable intake: (1) personal factors: knowledge, attitudes, liking, general self-efficacy, preferences, and perceived barriers (lack of time, liking sweets more, sticky fingers, ...), (2) perceived social-environmental factors: modeling, active parental encouragement, family rules – demands (whether parents demand their child to eat vegetables) and allowances (whether parents allow their child to eat as much vegetables as they want to) -, and parental facilitation, (3) perceived physical-environmental factors: availability at home, bringing fruit/vegetables to school, availability at school, and availability at friends' home. These constructs were assessed with 1 to 12 items, and for each construct a composite score was calculated as the mean of the relevant item scores. Responses were given on 5-point scales ranging from (-2) fully disagree/never to (+2) fully agree/always. An overview of the items, constructs and scaling are reported elsewhere [12,15]. A separate study in 5 countries showed sufficient internal consistencies for composite scores (alphas between 0.59 and 0.89), good to very good test-retest reliability (most ICC>0.60; all ICC>0.50) and moderate to good predictive validity (Spearman *r *ranging from 0.16 to 0.54 for personal factors, and from 0.05 to 0.38 for environmental factors) compared to other studies [15].

Usual fruit and vegetable intake was measured using a food-frequency questionnaire. Children were asked how often they usually eat fresh fruit, salad or grated vegetables, other raw vegetables and cooked vegetables. Response categories were (1) never, (2) less than one day per week, (3) one day per week, (4) 2–4 days a week, (5) 5–6 days a week, (6) every day, once a day, (7) every day, twice a day and (8) every day, more than twice a day. The sum of salad or grated, other raw or cooked vegetables was used to calculate usual vegetable intake as portions per day.

A separate study was executed to test the reliability and validity of these intake measures in 6 countries [15]. Results showed good test-retest reliability (Spearman *r *from 0.45 to 0.77), and adequate validity comparing the food-frequency questions with 7-day food records (Spearman *r *from 0.38 to 0.53).

The parents reported the child's height and weight and BMI's were calculated. International cut-offs for overweight and obesity in childhood have been suggested by Cole et al [16]. These provide age-related cut-offs per gender to be used in international prevalence studies. The children were then categorized as above or below the age-sensitive cut-off levels for overweight and obese. A potential confounder in the assessed associations can be the economic position of the child's family, which was estimated by the family educational level. Family educational level was assessed in the parents' questionnaire. The highest level of education reported by either parent was used as a combined estimate of family educational level.

### Statistical analysis

Differences in mean scores on the personal, social and environmental factors related to vegetable intake between the normal weight and overweight group were first analyzed using a general MANOVA. In addition independent *t *tests were performed to investigate differences in specific correlates. For these *t *tests, the p-value was set at 0.01 to balance between type 1 and type 2 errors for multiple testing on correlated variables [17].

Pearson correlations were computed between vegetable intake and the potential correlates specific for normal weight and overweight boys. A z-test was computed to compare the correlations between the normal weight and the overweight boys.

Bivariate correlations higher than 0.20 were selected to be entered in the regression analyses. Next, inter-correlations were computed between all selected predictors. For predictors showing inter-correlations higher than 0.50, only the predictor with the highest bivariate correlation with the criterion was kept, the others were removed from the model to reduce multicollinearity.

Different regression analyses were performed on the normal weight and overweight group. As the sample sizes used in the two regression analyses were very different (number of boys in the normal weight group about 6 times higher than number of boys in the overweight group) p-values, that depend heavily on sample size, cannot be compared. Standardized beta weights, and the semi-partial correlations were reported to make comparability of the regression models possible and to compare relative weights of the individual correlates. The multiple semi-partial correlations reflect the correlation between predictor and criterion controlling for all other predictors entered into the regression analyses. Therefore, the same predictors were included in both regression analyses. All analyses were executed on two samples: first on the total sample including all nine European countries of the Pro Children study, and secondly on the sub-sample of 4 countries (Austria, Belgium, Iceland, and Norway) with a clear negative association between vegetable intake and overweight. In the total sample, a significant relationship was found between family education level and overweight status (Chi^2 ^= 65.22, p > 0.001). The percentage of overweight boys was higher (28.6%) in families with the lowest educational level compared with the highest educational level (13.3%). Therefore analyses were adjusted for family education level. Further analyses were also adjusted for country using dummy coding.

All analyses were conducted in 2005 using SPSS 12.0.

## Results

### Differences in correlates of vegetable intake between overweight and normal weight boys

Overall MANOVA analyses with the psychosocial factors as dependent variables yielded significance (F(15,3452) = 2.89, p < 0.001). Mean scores for specific correlates are presented in table 1.

**Table 1:** Mean scores (SD) and Differences in Correlates of Vegetable Intake between Normal Weight and Overweight Boys

	**Total sample of all 9 countries (n = 3960)**			**Sub-sample of 4 countries (n = 1758)**		
	Normal weight boys (*n *= 3306)	Overweight boys (*n *= 654)	T-value (sign.)	Normal weight boys (*n *= 1493)	Overweight boys (*n *= 265)	T-value (sign.)

**Personal**						
Knowledge	0.24 (0.43)	0.27 (0.44)	-1.39	0.22 (0.42)	0.26 (0.44)	-1.20
Attitudes	0.82 (1.05)	0.87 (1.11)	-0.81	0.82 (1.02)	0.82 (1.08)	0.06
Liking	0.57 (1.14)	0.56 (1.21)	0.31	0.63 (1.15)	0.63 (1.24)	0.11
General self-efficacy	0.71 (1.05)	0.69 (1.07)	0.53	0.74 (1.06)	0.74 (1.07)	-0.04
Preferences	0.41 (0.87)	0.44 (0.92)	-0.86	0.53 (0.85)	0.57 (0.88)	-0.65
Perceived barriers	-1.08 (0.98)	-1.01 (1.10)	-1.49	-1.03 (1.00)	-1.01 (1.10)	-0.21
						
**Social-environmental**						
Modeling	0.63 (0.83)	0.64 (0.86)	-0.44	0.56 (0.82)	0.52 (0.80)	0.65
Active parental encouragement	0.28 (1.27)	0.35 (1.32)	-1.12	0.20 (1.21)	0.05 (1.31)	1.81
Demand family rule	0.11 (1.30)	0.12 (1.34)	-0.16	-0.18 (1.26)	-0.42 (1.27)	2.91*
Allow family rule	1.32 (1.02)	1.33 (1.03)	-0.32	1.33 (1.00)	1.41 (0.94)	-1.27
Family facilitation	-0.42 (1.31)	-0.37 (1.36)	-0.84	-0.50 (1.24)	-0.58 (1.26)	0.96
						
**Physical-environmental**						
Availability at home	0.99 (0.80)	1.04 (0.88)	-1.26	0.89 (0.81)	0.88 (0.88)	0.15
Bring vegetables to school	-1.22 (1.12)	-1.28 (1.12)	1.35	-1.18 (1.08)	-1.15 (1.09)	-0.30
Availability at school	-0.87 (1.49)	-1.01 (1.46)	2.29	-1.00 (1.38)	-1.11 (1.33)	1.16
Availability at friends house	-0.24 (1.30)	-0.49 (1.33)	4.39**	-0.12 (1.27)	-0.32 (1.26)	2.37*

* p < 0.01 ** p < 0.001

In the total sample of nine countries there was only one significant difference in correlates of vegetable intake between normal weight and overweight boys. Boys in the overweight group reported less availability of vegetables at their friends' house compared with normal weight boys. In the sub-sample of the four countries where a negative relationship between vegetable intake and overweight was found in boys, analyses revealed one additional significant factor. Overweight boys reported less demand from their parents to eat vegetables compared with normal weight boys.

### Correlations between vegetable intake and personal, social and environmental correlates in overweight and normal weight boys

Pearson correlations between vegetable intake and personal, social and environmental correlates were computed separately for overweight and normal weight boys. For personal factors, highest correlations were found for preferences, liking and self-efficacy (r between .30 and .40). Analyses showed somewhat lower correlations for knowledge, attitudes and perceived barriers. However, few differences were found in the strength of the correlations between normal weight and overweight boys. Correlations between social factors and vegetable intake were all between .20 and .30, except for 'the allow family rule' (i.e. if parents allowed their child to eat vegetables whenever he/she wants) which showed lower correlations. For active parental encouragement, demand family rule and family facilitation, the correlations were somewhat higher in the overweight boys compared to the normal weight boys. This means that parental encouragement, demand and facilitation appear to be somewhat stronger related to vegetable intake in overweight boys. The z-test only showed a trend towards significance (p = 0.07) for active parental encouragement. Correlations between physical-environmental factors and vegetable intake were highest for availability at home and bringing vegetables to school. Low correlations were found for availability of vegetables at friends' house and at school. Correlations between availability at home and vegetable intake were somewhat stronger in overweight boys compared to normal weight boys, which indicates that availability at home is more important for vegetable intake in overweight boys. The Pearson correlations between vegetable intake and personal, social and environmental correlates showed the same pattern in the full sample and in the sub-sample of four countries. Only for knowledge a marked difference was observed as a weaker association was seen for this variable among overweight boys in the sub-sample compared to overweight boys in the full sample.

### Prediction of vegetable intake by personal, social and environmental correlates in normal and overweight boys

Three correlates were deleted from the predictor list before running the regression analyses because bivariate correlations with vegetable intake were below .20: the allow family rule, availability of vegetables at school and availability at friends' house. As inter-correlations between general attitudes, preferences and liking were too high, only liking was included in the regressions. Similarly, high inter-correlations were found between active parental encouragement and the demand family rule; and only parental encouragement was included in the regressions.

The regression analyses for the normal weight and the overweight boys are presented in table 2 including all nine countries and in table 3 including the four countries with a negative association between vegetable intake and overweight status. Total explained variance was 23% for both the normal weight and the overweight group in the total sample. The explained variance was somewhat higher in the sub-sample of four countries: 25% in the normal weight group and 28% in the overweight group.

**Table 2:** Regression Analysis Predicting Vegetable Intake from Personal, Social and Environmental Correlates in Normal Weight And Overweight Boys in the total sample of all 9 countries (n = 3960)

**Normal weight group**				**Overweight group**			
***Potential correlates***	***Unstand. Coeff. B***	***Stand. Coeff. Beta***	***Semi-partial corr***.	***Potential correlates***	***Unstand. Coeff. B***	***Stand. Coeff. Beta***	***Semi-partial corr***.

*Total Model (F(9,3083) = 90.16)*				Total Model (F(9,597) = 18.69)			
*Adjusted R*^*2 *^= .*23*				*Adjusted R*^*2 *^= .*23*			
							
**Personal**				**Personal**			
Knowledge	0.29	0.13	0.12	Knowledge	0.20	0.09	0.09
Liking	0.13	0.15	0.11	Liking	0.12	0.15	0.12
General self-efficacy	0.10	0.10	0.08	General self-efficacy	0.12	0.14	0.11
Perceived barriers	-0.04	-0.04	-0.03	Perceived barriers	0.00	0.00	0.00
**Social-environmental**				**Social-environmental**			
Modeling	0.13	0.11	0.09	Modeling	0.06	0.05	0.05
Active parental encouragement	0.06	0.08	0.07	Active parental encouragement	0.11	0.16	0.13
Family facilitation	0.05	0.06	0.06	Family facilitation	0.05	0.07	0.06
**Physical-environmental**				**Physical-environmental**			
Availability at home	0.06	0.05	0.04	Availability at home	0.13	0.12	0.10
Bring vegetables to school	0.11	0.12	0.12	Bring vegetables to school	0.05	0.06	0.05

Analyses were adjusted for family education level

**Table 3:** Regression Analysis Predicting Vegetable Intake from Personal, Social and Environmental Correlates in Normal Weight And Overweight Boys in the sub-sample of 4 countries (Austria, Belgium, Iceland, and Norway) (n = 1758)

**Normal weight group**				**Overweight group**			
***Potential correlates***	***Unstand. Coeff. B***	***Stand. Coeff. Beta***	***Semi-partial corr***.	***Potential correlates***	***Unstand. Coeff. B***	***Stand. Coeff. Beta***	***Semi-partial corr***.

*Total Model (F(12,1394) = 36.77)*				*Total Model (F(12,227) = 7.61)*			
*Adjusted R*^*2 *^= .*25*				*Adjusted R*^*2 *^= .*28*			
							
**Personal**				**Personal**			
Knowledge	0.28	0.12	0.12	Knowledge	0.02	0.01	0.01
Liking	0.12	0.14	0.10	Liking	0.08	0.14	0.10
General self-efficacy	0.08	0.09	0.07	General self-efficacy	0.09	0.13	0.10
Perceived barriers	-0.07	-0.07	-0.06	Perceived barriers	0.00	0.00	0.00
**Social-environmental**				**Social-environmental**			
Modeling	0.14	0.12	0.10	Modeling	0.03	0.03	0.03
Active parental encouragement	0.03	0.04	0.03	Active parental encouragement	0.07	0.13	0.11
Family facilitation	0.04	0.05	0.04	Family facilitation	0.02	0.03	0.02
**Physical-environmental**				**Physical-environmental**			
Availability at home	0.07	0.06	0.05	Availability at home	0.16	0.19	0.15
Bring vegetables to school	0.16	0.18	0.16	Bring vegetables to school	0.12	0.17	0.15

Analyses were adjusted for country and for family education level

In the normal weight group, knowledge about the vegetable intake recommendations, liking vegetables a lot, general self-efficacy, modeling, and bringing vegetables to school showed the strongest associations with vegetable intake (Beta > .10). Beta's were similar in the total sample and the sub-sample. In the overweight group, liking vegetables and general self-efficacy were also among the factors most strongly related to vegetable intake. However, in contrast to normal weight boys, active parental encouragement and availability at home were significant correlates of vegetable intake among overweight boys (Beta > .10), while the knowledge of the recommendation, modeling, and bringing vegetables to school appeared to be of less importance (Beta < .10). In the sub-sample, bringing vegetables to school showed up to be an important predictor of vegetable intake among both normal weight and overweight boys.

## Discussion

Overweight children have been found to have different dietary intakes then their normal weight peers [2,18], but it is less clear if overweight children differ in potential determinants of intakes. The present study aimed to investigate the association of vegetable intake in boys with a comprehensive set of possible correlates in a cross-national sample, including nine European countries, according to overweight status. The study is unique in its exploration of potential determinants of intake dependent on weight status.

Only few differences were found in personal, social-environmental, and physical-environmental correlates of vegetable intake between normal weight and overweight boys. Overweight boys report less demands from their parents to eat vegetables every day. It is possible that this is an expression of the general parenting style often found in parents of overweight children. They tend to be less demanding and less strict in setting and following rules in general [19]. Another possibility is that parents of overweight children have learnt not to force their children anymore to eat, as they are already overweight. Nonetheless, in overweight children, it could be a good intervention strategy that parents insist as far as possible that their children eat a lot of foods with low energy-density, such as vegetables every day to promote energy balance [4,5,20]. However, there is still inconsistency in the literature as to whether parental demanding of eating healthy foods or restricting unhealthy foods leads to more healthy [21] versus more unhealthy eating habits [22]. A second difference was found for the availability of vegetables at friends' house. Although both normal weight and overweight boys reported low availability of vegetables at friends' house in general, reports were even lower in the overweight boys. It is possible that overweight boys have overweight friends which consume few vegetables, or that overweight boys just have less friends where they stay to eat [23,24].

The regression analyses also revealed only few differences in strength for the potential correlates according to overweight status. Two correlates were stronger for overweight boys than for normal weight boys: active parental encouragement and availability at home. More encouragement of parents to eat vegetables regularly was associated with higher intake levels in overweight boys. In normal weight boys, this association was very weak, but a stronger modeling effect was found. Home environment is know to influence childhood obesity, and similarities between parent and child are known for physical activity and obesity [25,26]. This study indicates that it is possible that overweight boys need more active encouragement strategies to impact upon their intake, while in normal weight boys modeling might be enough. Previous studies already showed the association between parental intake of vegetables and children's consumption as well as the impact of parental control, support, and encouragement upon vegetable intake [27-30]. However, no study previously revealed that differences in strength of these predictors might be related to weight status. From the present study it could be hypothesized but not yet proven that advising parents of overweight boys to be more 'active' in encouraging their children could have a positive effect on their daily vegetable intake. In addition, the lower reports of overweight boys of parental demand to eat vegetables every day, together with the considerable correlation between these parental demands and vegetable intake in overweight boys, further corroborate these suggestions.

The present study revealed that the earlier established relationship between home availability and intake [26,30-32] was especially apparent in overweight boys. This means that increasing home availability, for example by buying vegetables that the child likes, by buying different kinds of vegetables, and by buying the vegetables the child asks for, might be especially important for them.

In contrast, the relationship between knowledge of the recommendations and vegetable intake [27,32-35] was *weaker *in overweight boys compared to normal weight boys, suggesting that increasing knowledge of recommended intake levels will have less impact in overweight boys.

There are several limitations to the present study. First, the cross-sectional nature of the study does not allow prediction, nor conclusions about how much change in correlates is predictive of change in vegetable intake. A reciprocal relationship between the correlates and consumption may also be likely. This study does not say anything about the reasons for weight gain and development of overweight. A different research approach is necessary to investigate the behaviour, and its determinants, leading to overweight and obesity. Second, Body Mass Index was calculated based on self-reported weight and height from parents, which could have been resulted in some underreporting of (over)weight [36, 37, 38, 39, 40] or in lack of knowledge of the weight and height of their child, given the velocity of growth at this age. In large population samples, direct measurement is often not possible. However Strauss [38, 41] showed that self-reported weight and height can be used in large samples, because these self-reported measures resulted in the correct classification of weight status in 94% of their adolescent population, which has been used in other large epidemiological studies [42]. Third, all other data are also based on self-reports of behavior and correlates. The Pro Children study applied a standardised, validated instrument to measure fruit and vegetable intake and its potential psychosocial correlates. However, the questionnaire contained more than 100 items, and was quite demanding to respond to for such young children. As the validity and the test-retest reliability was good for almost all constructs we believe that children of this age were able to respond in a meaningful and reliable way. Finally, in the present study, we only investigated potential correlates of vegetable intake in overweight and normal weight boys. As previous analyses showed no relationship between overweight and vegetable intake in girls or between overweight and fruit intake in boys and girls [7], analyzing potential correlates in these subgroup related to overweight was not relevant. However, additional research is needed about the relationship between fruit and vegetable intake and weight status in this age group.

Important strengths of the study are its large international sample, and the use of standardised, validated instrument to measure fruit and vegetable intake and its potential psychosocial and environmental correlates across diverse food-related cultural settings. Country samples are representative, total sample size is large, and we obtained a high participation rate.

## Conclusion

The present study suggests that intervention strategies to increase vegetable intake in normal weight and overweight 11 year old boys can tackle partly the same factors for both groups. Focusing on mediators such as increase in liking and preferences, increase in self-efficacy, and increase in bringing vegetables to school might result in higher vegetable consumption levels in normal weight and overweight boys. However, it could be hypothesized that parents of overweight boys that give specific attention to encourage their children to eat vegetables every day, to insist as far as possible that their child eats vegetables regularly and to make different kinds of vegetables that their child likes and asks for, available at home, would have an important impact upon the vegetable consumption of their sons.

## Competing interests

The author(s) declare that they have no competing interests.

## Authors' contributions

IDB conceived of the study, and directed all aspects of the study including development, assessment and analyses, and lead the writing of the manuscript. AY, StV, KIK, MR, IT, AW, JB all helped to develop measures, conduct assessment and analyses and assisted with the writing of the manuscript. All authors read and approved the final manuscript.

## References

[B1] (2000). Obesity: preventing and managing the global epidemic. Report of a WHO consultation. World Health Organ Tech Rep Ser.

[B2] Lobstein T, Baur L, Uauy R (2004). Obesity in children and young people: a crisis in public health. Obes Rev.

[B3] (2004). WHO Global Strategy on Diet, Physical Activity and Health - what are the challenges for follow-up in Europe?: Workshop 1. Eur J Public Health.

[B4] Tohill BC, Seymour J, Serdula M, Kettel-Khan L, Rolls BJ (2004). What epidemiologic studies tell us about the relationship between fruit and vegetable consumption and body weight. Nutr Rev.

[B5] Rolls BJ, Ello-Martin JA, Tohill BC (2004). What can intervention studies tell us about the relationship between fruit and vegetable consumption and weight management?. Nutr Rev.

[B6] Yngve A (2005). Intake of fruit and vegetables in European children and their mothers, folate intake in Swedish children and health indicators - overweight, plasma homocysteine levels and school performance. Department of biosciences and nutrition.

[B7] Klepp KI, Perez-Rodrigo C, De Bourdeaudhuij I, Due PP, Elmadfa I, Haraldsdottir J, Konig J, Sjostrom M, Thorsdottir I, Vaz de Almeida MD, Yngve A, Brug J (2005). Promoting fruit and vegetable consumption among European schoolchildren: rationale, conceptualization and design of the pro children project. Ann Nutr Metab.

[B8] Yngve A, Wolf A, Poortvliet E, Elmadfa I, Brug J, Ehrenblad B, Franchini B, Haraldsdottir J, Krolner R, Maes L, Perez-Rodrigo C, Sjostrom M, Thorsdottir I, Klepp KI (2005). Fruit and vegetable intake in a sample of 11-year-old children in 9 European countries: The Pro Children Cross-sectional Survey. Ann Nutr Metab.

[B9] Patrick H, Nicklas TA (2005). A review of family and social determinants of children's eating patterns and diet quality. J Am Coll Nutr.

[B10] Rasmussen M, Krolner R, Klepp KI, Lytle L, Brug J, Bere E,  Due P (2006). Determinants of fruit and vegetable consumption among children and adolescents: systematic review of the literature. Part I: quantitative studies. Int J Behav Nutr Phys Act.

[B11] Wind M, Bobelijn K, De Bourdeaudhuij I, Klepp KI, Brug J (2005). A qualitative exploration of determinants of fruit and vegetable intake among 10- and 11-year-old schoolchildren in the low countries. Ann Nutr Metab.

[B12] Sandvik C, De Bourdeaudhuij I, Due P, Brug J, Wind M, Bere E, Perez-Rodrigo C, Wolf A, Elmadfa I, Thorsdottir I, Vaz de Almeida MD, Yngve A, Klepp KI (2005). Personal, social and environmental factors regarding fruit and vegetable intake among schoolchildren in nine European countries. Ann Nutr Metab.

[B13] De Bourdeaudhuij I Personal, social and environmental correlates of daily fruit and vegetable intake in 11-year old children in 9 European countries.

[B14] Wolf A, Yngve A, Elmadfa I, Poortvliet E, Ehrenblad B, Perez-Rodrigo C, Thorsdottir I, Haraldsdottir J, Brug J, Maes L, Vaz de Almeida MD, Krolner R, Klepp KI (2005). Fruit and vegetable intake of mothers of 11-year-old children in nine European countries: The Pro Children Cross-sectional Survey. Ann Nutr Metab.

[B15] De Bourdeaudhuij I, Klepp KI, Due P, Rodrigo CP, de Almeida M, Wind M, Krolner R, Sandvik C, Brug J (2005). Reliability and validity of a questionnaire to measure personal, social and environmental correlates of fruit and vegetable intake in 10-11-year-old children in five European countries. Public Health Nutr.

[B16] Cole TJ, Bellizzi MC, Flegal KM, Dietz WH (2000). Establishing a standard definition for child overweight and obesity worldwide: international survey. Bmj.

[B17] Barton BA, Eldridge AL, Thompson D, Affenito SG, Striegel-Moore RH, Franko DL, Albertson AM, Crockett SJ (2005). The relationship of breakfast and cereal consumption to nutrient intake and body mass index: the National Heart, Lung, and Blood Institute Growth and Health Study. J Am Diet Assoc.

[B18] Brann LS, Skinner JD (2005). More controlling child-feeding practices are found among parents of boys with an average body mass index compared with parents of boys with a high body mass index. J Am Diet Assoc.

[B19] Lanza E, Schatzkin A, Daston C, Corle D, Freedman L, Ballard-Barbash R, Caan B, Lance P, Marshall J, Iber F, Shike M, Weissfeld J, Slattery M, Paskett E, Mateski D, Albert P (2001). Implementation of a 4-y, high-fiber, high-fruit-and-vegetable, low-fat dietary intervention: results of dietary changes in the Polyp Prevention Trial. Am J Clin Nutr.

[B20] Eisenberg ME, Neumark-Sztainer D, Story M (2003). Associations of weight-based teasing and emotional well-being among adolescents. Arch Pediatr Adolesc Med.

[B21] Eisenberg ME, Neumark-Sztainer D, Story M, Perry C (2005). The role of social norms and friends' influences on unhealthy weight-control behaviors among adolescent girls. Soc Sci Med.

[B22] Strauss RS, Knight J (1999). Influence of the home environment on the development of obesity in children. Pediatrics.

[B23] Fogelholm M, Nuutinen O, Pasanen M, Myohanen E, Saatela T (1999). Parent-child relationship of physical activity patterns and obesity. Int J Obes Relat Metab Disord.

[B24] Hanson NI, Neumark-Sztainer D, Eisenberg ME, Story M, Wall M (2005). Associations between parental report of the home food environment and adolescent intakes of fruits, vegetables and dairy foods. Public Health Nutr.

[B25] Bere E, Klepp KI (2004). Correlates of fruit and vegetable intake among Norwegian schoolchildren: parental and self-reports. Public Health Nutr.

[B26] Woodward DR, Boon JA, Cumming FJ, Ball PJ, Williams HM, Hornsby H (1996). Adolescents' reported usage of selected foods in relation to their perceptions and social norms for those foods. Appetite.

[B27] Neumark-Sztainer D, Wall M, Perry C, Story M (2003). Correlates of fruit and vegetable intake among adolescents. Findings from Project EAT. Prev Med.

[B28] Cullen KW, Baranowski T, Owens E, Marsh T, Rittenberry L, de Moor C (2003). Availability, accessibility, and preferences for fruit, 100% fruit juice, and vegetables influence children's dietary behavior. Health Educ Behav.

[B29] Reynolds KD, Baranowski T, Bishop DB, Farris RP, Binkley D, Nicklas TA, Elmer PJ (1999). Patterns in child and adolescent consumption of fruit and vegetables: effects of gender and ethnicity across four sites. J Am Coll Nutr.

[B30] Osler M, Hansen ET (1993). Dietary knowledge and behaviour among schoolchildren in Copenhagen, Denmark. Scand J Soc Med.

[B31] Bere E, Klepp KI (2005). Changes in accessibility and preferences predict children's future fruit and vegetable intake. Int J Behav Nutr Phys Act.

[B32] Kratt P, Reynolds K, Shewchuk R (2000). The role of availability as a moderator of family fruit and vegetable consumption. Health Educ Behav.

[B33] Brener ND, McManus T, Galuska DA, Lowry R, Wechsler H (2003). Reliability and validity of self-reported height and weight among high school students. J Adolesc Health.

[B34] Andersen LF, Lillegaard IT, Overby N, Lytle L, Klepp KI, Johansson L (2005). Overweight and obesity among Norwegian schoolchildren: changes from 1993 to 2000. Scand J Public Health.

[B35] Strauss RS (1999). Comparison of measured and self-reported weight and height in a cross-sectional sample of young adolescents. Int J Obes Relat Metab Disord.

[B36] Boutelle K, Fulkerson JA, Neumark-Sztainer D, Story M (2004). Mothers' perceptions of their adolescents' weight status: are they accurate?. Obes Res.

[B37] Jackson J, Strauss CC, Lee AA, Hunter K (1990). Parents' accuracy in estimating child weight status. Addict Behav.

[B38] Goodman E, Strauss RS (2003). Self-reported height and weight and the definition of obesity in epidemiological studies. J Adolesc Health.

[B39] Taveras EM, Berkey CS, Rifas-Shiman SL, Ludwig DS, Rockett HR, Field AE, Colditz GA, Gillman MW (2005). Association of consumption of fried food away from home with body mass index and diet quality in older children and adolescents. Pediatrics.

